# Do research experience programs promote capacity building in Qatar: Investigating the trend and participation differences

**DOI:** 10.1016/j.heliyon.2023.e22071

**Published:** 2023-11-04

**Authors:** Zubair Ahmad, Mohammad Ammar, Nitha Siby, Jolly Bhadra, Abdellatif Sellami, Noora J. Al-Thani

**Affiliations:** a Qatar University Young Scientists Center (QUYSC), Qatar University, 2713, Doha, Qatar; b College of Education, Qatar University, 2713, Doha, Qatar

**Keywords:** Undergraduate research experience, Middle east, Sustainable development, STEM education

## Abstract

Research Experience programs (REPs) inspire students to pursue advanced degrees and shape their research career paths. Government and commercial organizations sponsor REPs to promote the capacity building of the country. In Qatar, the national youth is reported to show concerning participation in Science, Technology, Engineering, and Mathematics (STEM) disciplines at the K-12 level. However, none of the studies investigate these participation trends at the undergraduate level, especially in scientific research, which is deemed necessary for building a knowledge-based economy in Qatar. Therefore, to bridge this gap, the current study uses a quantitative approach to analyze the REP in Qatar through the participation data of 2455 undergraduate students. For this, statistical measures, including descriptive analysis, independent samples *t*-test, and Pearson's correlation analysis were used. Results indicated concerning trends in national student participation rate, implying underlying issues restricting their representation in undergraduate research activities. Also, statistically significant differences were found in student participation rates among students' gender and ethnic distributions. While female students demonstrated higher participation rates than males, national students showed lower participation than the non-nationals. Moreover, this low participation of national students suffered more drastically in STEM disciplines. Therefore, these findings determine the outlook for stakeholders and academic institutions in making meaningful educational decisions and envision synchronizing REPs at the university level, gauging measures to bolster the adjacent funding agencies and government organizations. Furthermore, being the first research addressing REPs in the Middle East region, this study has the potential to support educators in neighboring and other developing nations where STEM education is especially significant for human capacity building.

## 1 Introduction

Undergraduate research experiences influence students in pursuing graduate studies, leading to professional accomplishments in their higher education. These transformational experiences help students transition from knowledge-based learning to application-based hands-on training [1]. These experiences also nurture students’ grasp of competent professional skills, including analytical and critical thinking, problem-solving, and research-oriented innovational thinking [2]. Nourishing these essential skills eventually contributes to sustainable progress and development.

Indeed, many countries invest a significant amount of their Gross Domestic Product (GDP) into Research and Development (R&D) to meet their sustainable development needs. For instance, according to the United Nations Educational, Scientific and Cultural Organization (UNESCO) [3], South Korea and Japan spend 4.1 % and 3.4 % of their GDP on research, respectively. Germany invests more than 2.8 % of its GDP in research, while the United States (US) spends 2.7 %. As part of their R&D policy agendas, these countries seek to attract foreign researchers and skilled immigrants by granting them permanent residencies and citizenship. Interestingly, this contrasts sharply with the current state of affairs in Arab countries, which do not offer any naturalization laws that allow foreigners, including highly skilled professionals, to gain permanent residency or citizenship. This is also the case in Qatar, where the expatriate students know that long-term plans of staying and working in Qatar are not a possible option for them. Therefore, Qatar must rely on and stress the importance of its local talent.

Qatar is a dynamic and forward-thinking nation in the heart of the Arabian Gulf that has risen in recent years as a beacon of scientific innovation [4]. Qatar has invested extensively in R&D, establishing itself at the forefront of scientific discoveries as part of its strong commitment to developing knowledge and technology. The forefront of this R&D innovation transformation began in 2012 when the nation's leaders established the Qatar National Research Strategy (QNRS2012) to enhance the merits and abilities of individuals as well as institutions inside Qatar [5]. This laid the ground for the country to create a dynamic atmosphere conducive to groundbreaking research in a variety of subjects. Qatar's commitment to scientific progress sees further progress with its current strategic national development plan outlined by the Qatar National Vision (QNV2030), seeking to raise the R&D profile of the country by extensively focusing on four strategic pillars: Energy and Environment; Computer Science and ICT; Health; and Social Sciences, Arts and Humanities [6].

However, a major concern for Qatar is the low interest of national students in pursuing Science, Technology, Engineering, and Mathematics (STEM) related career paths [7]. This issue becomes more severe as the country's goals of the QNV2030 encourage the positive attitudes of national students towards STEM-related fields. Further, the QNV2030 aims to establish a countrywide network of informal educational initiatives that stimulate innovation and creativity and give the national youth the tools and incentives to contribute to society. These goals will give Qatar a vital role globally in intellectual, artistic, and scientific pursuits. For these reasons, it is equally important to analyze the involvement of national students of Qatar in Undergraduate Research Experience Programs (UREPs) as a measure of their interest in and inclination toward scientific research.

To achieve the strategic national goals of sustainable human capacity and develop local Research, Development, and Innovation (RDI) talents, the UREP practices have been progressively implemented by Qatar National Research Fund (QNRF) since 2006 [8,9]. UREP aims to develop adequate research infrastructure in Qatar by establishing a solid foundation for scientific research at the Undergraduate (UG) level. Over the past fifteen years, UREP has stimulated a broad array of UG research initiatives, supplementing existing UG research opportunities in local academic institutions and contributing to workforce training by funding research activities beyond those offered via regular coursework. No doubt, QNRF plays a crucial role in promoting a research culture within society by awarding research grants consistently for promising research across a wide range of disciplines. The organization showed a keen interest in fostering home-grown researchers and scientists, thereby ensuring financial assistance for projects that could yield positive results. This initiative has catalyzed the promotion of research within the country, offering student grants and fellowships to researchers at the pre-college, post-secondary, and tertiary levels of education.

However, as we reeled into the literature on prior studies assessing the involvement of national students in research engagement, the low participation of national students in research was apparent, though there were limited evidence-based surveys. Even though a few survey studies were performed on science trends within the national student body at the K-12 level [10,11], these studies are slightly outdated. None of the studies inform readers about the interest of national students at the UG level, specifically in scientific research, which is deemed necessary for building a knowledge-based economy in Qatar.

Our study primarily aimed to analyze the participation levels of National Undergraduate (NUG) students in UREP in terms of national capacity building. To this end, a detailed analysis of the data concerning the yearly participation trends of students is conducted, inspecting their ethnic and gender-based differences in different research disciplines. The scope of this study is to understand the current participation trend and differences of UREP in Qatar and identify the areas that require immediate attention. The analysis provides insights for decision-makers to improve the learning environment of tertiary (college and university) students through various institutional reforms. Overall, this study answers the following research questions:

(1) How are the participation trends of national students in undergraduate research in Qatar?
(2) Are there any significant differences in student participation in undergraduate research in Qatar?
(3) How do these differences reflect in the STEM disciplines of undergraduate research at Qatar?

## 2 Literature review

Throughout history, research apprenticeships have been the dominating model used to provide out-of-class education to graduate students [12]. However, over the last two decades, apprenticeship experiences for secondary and UG students have been more frequently developed [13]. Students' authentic research experiences are facilitated through institutionalized and structured programs that effectively help mentor students' experiences. These programs provide students a chance to participate in the process of scientific discovery and allow them a better understanding of what a research career can involve. They gain the capacity to “think like a scientist,” change their perspectives towards education, and function as researchers and individuals [14,15]. Furthermore, it is reported that students who participate in research early in their careers are more inclined to remain in STEM professions, with first-generation students showing the most significant improvements [16]. Therefore, UG research is a particularly effective tool for motivating and assisting students in STEM fields, and student involvement in such research raises their engagement, commitment, and graduation rates in these fields. However, studies indicate that UG research experiences are crucial for the growth and maintenance of UGs’ interests in STEM careers across various age groups, program frameworks, intensities, and durations [17]. Nevertheless, the style adopted by research program mentors to describe scientific procedures and explain the use of equipment may majorly affect both the rate and depth of such learning [18].

In the US, this idea of the intrinsic significance of student involvement in producing information instead of just consuming it gave rise to a larger push to boost UG research participation. Advocated by the US-based Council on UG Research, this led to the formation of the Undergraduate Research Opportunity Program (UROP) [19]. The purpose of these experiences was to improve students' research abilities while advancing academic staff members’ work. Having originated in the US, similar programs are now implemented in universities worldwide. However, complications occur when some students are underrepresented and cannot benefit from such research experiences, thus affecting the scientific literate citizenry within the STEM professional community [20]. Much attention in the literature has been focused on opportunities for UGs in STEM to develop the interests of underrepresented communities and expand their aspiration to pursue research apprenticeships [[21], [22], [23]].

Furthermore, the underrepresentation of national students in Qatar has been previously studied to highlight the importance of their representation in the STEM disciplines for the economic prosperity of the country [10,11,[24], [25], [26], [27], [28]]. The conclusions from these studies can be attributed to national students' interest, attitude, and self-efficacy levels in STEM areas, which can dependently increase their participation in pursuing science-related careers. The strong and commonly effective route to achieving this goal is through the effective involvement of national students in research experiences at the UG levels. Moreover, though studies on UG research have been reported from the Gulf region assessing students' research skills [29], attitudes [30,31], and barriers to student participation [32], no literature reported students’ participation and engagement in UG research based on ethnicity and gender differences.

Therefore, this paper focuses on addressing this specific concern in Qatar. The present findings have implications for REPs in neighboring Gulf nations or developing countries worldwide. Moreover, the results of this study call for future research to identify the measure to employ compelling UG research experiences with the representation of all communities, thus building a strong STEM workforce.

## 3 Methods

### 3.1 Data collection

A convenience sampling strategy was used in this study by obtaining data from the Office of Academic Research (OAR) at Qatar University (QU). To analyze the participation trends of students in UREPs at QU, the raw data was separated based on gender, ethnicity, cycle, and research discipline. This data ranged from 2006 (when UREP commenced) to 2020. Since UREP projects are awarded to institutions in cycles, the data were classified into 26 cycles. Gender and nationality information was based on students’ self-reports on the UREP applications. Further, admission and graduation data were also obtained from OAR. Approval was obtained from OAR at QU.

This sample data of UREP participation of students from QU represented the larger sample of UREP participation in Qatar. To confirm this, preliminary data was extracted from QNRF's online searchable database [33]. The primary details for the relevant projects from each cycle, included the project number, the project title, the lead investigator, the project status, the submitting institution, primary and secondary research areas, sub-research areas, subspecialty areas, the name and affiliation of each mentor, and the student(s) involved in the project. Gender was classified into male and female by identifying the prefix titles used for their names (Mr., Ms., and Mrs.). Students were categorized into nationals and expatriates with the help of local experts by identifying the surnames of students. Furthermore, it is worth noting that the data used in this study from QU is representative of the entire population of UG students in Qatar. Evidenced by Qatar's Planning and Statistics Authority, 2019 [34], QU has the distinction of accounting for 66.8 % of the total UG student population in the country, which is an acceptable representation.

To verify the accuracy of these data and obtain missing information, the extracted information from QNRF provided all the required information, including the number of projects awarded and discipline-wise participation of national and expatriates, males and females, respectively. Table S1 shows the division of sub-research areas examined under each immediate research area. It is to be noted here thatthe data from QNRF includes the data points for projects that were terminated in the future or canceled for whatever reasons, OAR did not consider such projects in its data. However, these differences are not a concern in this current study since the conclusions reported in this study are only based on the data provided by OAR. The data from QNRF merely supports the sample size data from OAR and provides references for our research.

### 3.2 Data cleaning and preparation

The obtained data from OAR was primarily used for all the analyses in this study. The data in its raw form was stored methodologically in Excel. Data cleaning was a crucial step in ensuring the reliability of our analysis. Our primary goal was to identify and address missing values, errors, and inconsistencies within the dataset. Missing data points were identified and imputed using mean imputation for numerical variables and mode imputation for categorical variables. Further, we searched for duplicate entries based on unique participant identifiers to address potential duplication within the dataset. Any duplicate entries identified were cross-verified with the original data source, and the most recent or accurate entry was retained, while duplicate entries were removed. These comprehensive data cleaning steps aimed to enhance the accuracy and reliability of the dataset, laying a robust foundation for the subsequent statistical analyses and interpretation of results.

### 3.3 Data analysis

First, the descriptive statistics of the sample data and overall UREP population concerning gender, ethnicity, and discipline-based factors were examined. The sample size of UREP participants from QU well-represented the overall population of UREP participants. The sample size accounts for more than half of the overall participants (53.1 %) and awarded projects (59.6 %). Moreover, descriptive statistics showed a high concordance between the distribution of both data sources based on gender, ethnicity, and discipline (see Table 1 and Table S2). Hence, the sample size data obtained from QU was deemed reliable for further analyses, confidently representing the overall population.

**Table 1:** Descriptive statistics of students from QU involved in UREPs from 2006 to 2020.

Variable	Sub-categories	Number of Participants (N = 2455)	Percentage of Participants	Number of Projects (N = 668)	Percentage of Projects
Gender	Male	766	31.2	–	–
	Female	1689	68.8	–	–
Ethnicity	Non-National	1736	70.7	–	–
	National	719	29.3	–	–
Regionality	Asia	1886	76.8	–	–
	Africa	507	20.7	–	–
	North America	36	1.5	–	–
	Europe	16	0.7	–	–
	Australia	10	0.4	–	–
Primary Research Area	Engineering & Technology	943	38.4	233	34.9
	Medical and Health Sciences	453	18.5	150	22.5
	Natural Sciences	473	19.3	136	20.4
	Social Sciences	415	16.9	110	16.5
	Humanities	134	5.5	28	4.2
	Agricultural Sciences	37	1.5	11	1.6
Discipline	STEM-related	1906	77.6	530	79.3
	Non-STEM related	549	22.4	138	20.7

Further, descriptive analyses were conducted, focusing on how gender and ethnicity-based factors affected the participation of students in UREPs. Admission and graduation rates classified according to gender and ethnicity were used to corroborate this trend of student participation in UREPs. Pearson's correlation tests [35] were performed to investigate and quantify any possible relation between variables that may conceivably contribute to student participation rates in UREPs. Pearson's correlation coefficient (also known as Pearson's r) was used, and its statistical significance was determined [36]. The Shapiro-Wilk's test was used to assess the normality of the distribution of demographic groups [37]. The significance of the test revealed normality at the 95 % confidence intervals (*p* > 0.05) for all distributions [38]. Therefore, an independent samples *t*-test was used to find statistical differences in participation rates between various demographic groups. IBM SPSS statistics v.28.0 was used for all the analyses.

## 4 Results

### 4.1 Descriptive analysis

Table 1 presents the descriptive statistics of the participants (*N* = 2455) of UREP from QU from 2006 to 2020. Participation from foreign students comprised 70.7 % of the total, and nationals comprised the remaining 29.3 %. The gender composition of the participants was about 68.8 % females and 31.2 % males. Further, discipline-based distribution yielded in the Engineering & Technology discipline involved 38.4 % of the participants, followed by Natural Sciences (19.3 %), Medical and Health Sciences (18.5 %), Social Sciences (16.9 %), Humanities (5.5 %), and Agriculture (1.5 %).

**Fig. 1:**
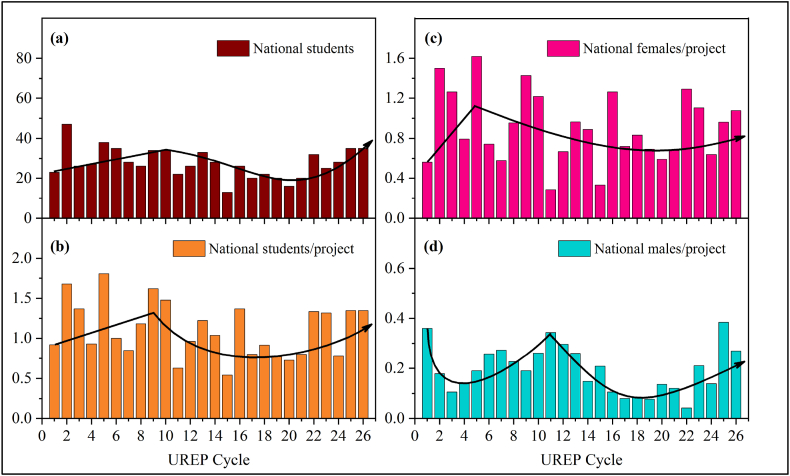
Propagation of NUG at QU through the UREP timeline. (a) Absolute numbers of national students (b) National students per project (c) National female students per project (d) National male students per project.

To analyze the participation rate of NUG students, the number of students per project was graphed against each cycle, as shown in Fig. 1(a–d). This depicts national students’ participation in each cycle, which is further classified into males and females for a better insight into gender-based trends in NUG students. It can be observed that the average number of male students per project reached a peak value of 0.36 during the first 10 cycles of UREP, which after that mainly decrements in regular intervals. Concerning females, the averages indicate that the ratio of female NUG students to UREP projects is 1:1, with the peak average during the first 5 cycles of UREP later decrementing to less than 1. In this regard, the QU embodies a worrisome situation, for there is a decreasing trend that continues over the different cycles. This is worrisome as QU is the only national university with the most significant number of NUG students admitted every year in the country.

**Fig. 2:**
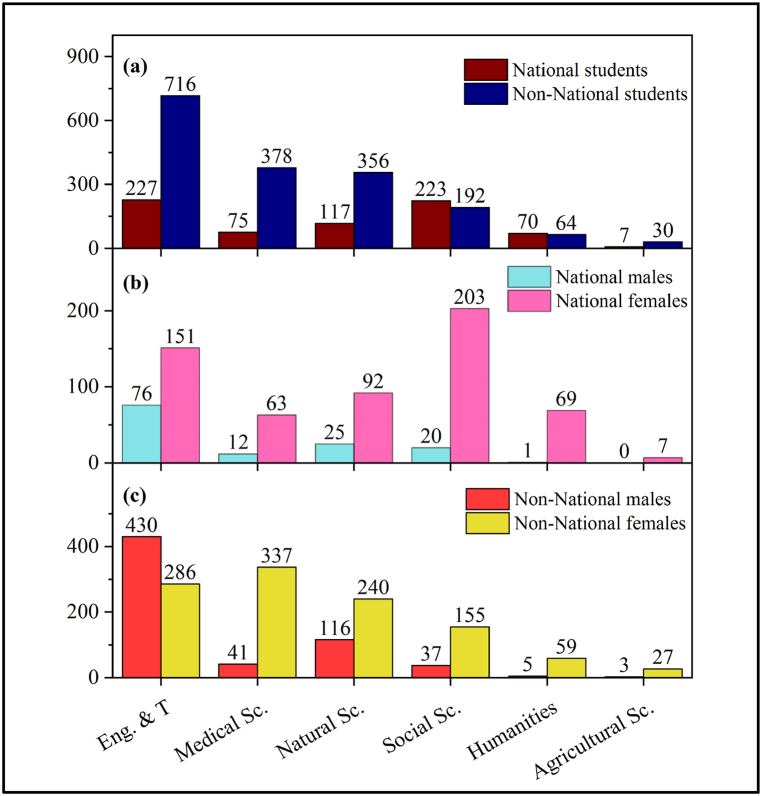
(a) Distribution of national and expatriate QU students in various primary research fields. (b) Distribution of male and female students among the national students who participated from QU in various primary research fields. (c) Distribution of male and female students among the expatriate students who participated from QU in various primary research fields.

Further, to compare the number of national and expatriate students in UREP based on discipline, the distribution of students is drawn against the research field of the UREP projects, as shown in Fig. 2. Here, it is interesting to observe that QU students dominate STEM disciplines. Data is splintered to further analyze student participation based on ethnicity and gender, as in Fig. 2(a–c). The detailed analysis in the graphs signifies that the foreign student population overwhelms national participation, except in non-STEM (Social Science and Humanities) disciplines. For, it is evident that STEM disciplines are significantly majored by expatriate participants. This observation further brings to attention a critical concern over the participation of males in the humanities discipline because their total involvement in UREP for the past 14 years from QU was meager (verified with data from OAR). Concerning the discipline of Agriculture Science, the situation is more worrying as the data points show that no single national male participation has ever been recorded. All the disciplines display an overwhelming presence of female students compared to their male counterparts. At the same time, within the foreign student population, many male students participated in the discipline of Engineering and technology, which is distinctive compared to the other research fields where the population of females is always more significant than the males.

**Fig. 3:**
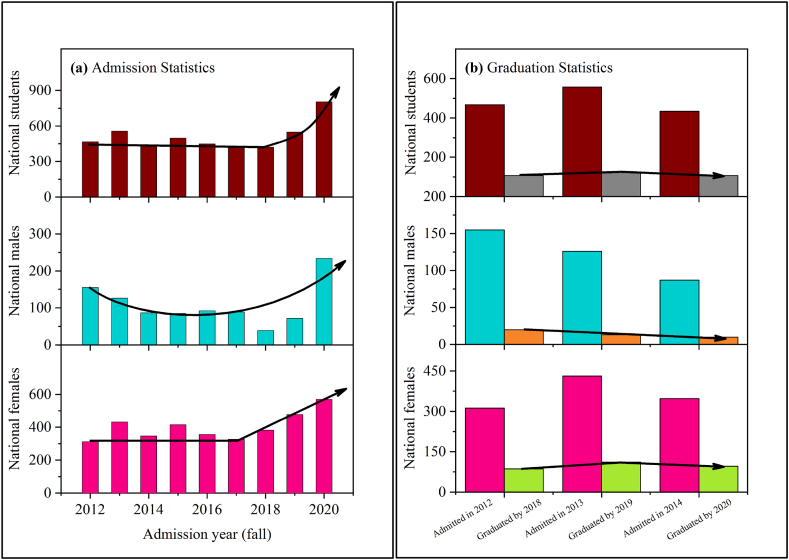
(a) Admission rate of national students in UG programs QU in STEM disciplines. (b) Graduate rate of national students in UG programs at QU in STEM disciplines.

To examine, “was the continuous lower participation of the NUG students from QU in UREP due to the less admission/enrollment of NUG students in STEM disciplines?” we analyzed NUG admission and graduation rates at QU (as shown in Fig. 3a and b). It was observed that the rate at which NUG students are admitted to STEM and other disciplines increased steadily during the last few years. However, the graduation rate of national male students was considerably low. The data presented in Fig. 3b demonstrates the average graduation period of six years (following discussions with the Student Affairs Department at QU). Hence, the students admitted in 2012 were supposed to graduate by 2018 and so on, with the reason ascribed to the varying course durations (4 or 5 years) in different disciplines at QU. In addition, some students were required to possess the prerequisites for specific courses and are thus obliged to take foundation courses before their degree programs, eventually graduating within 6 years.

**Fig. 4:**
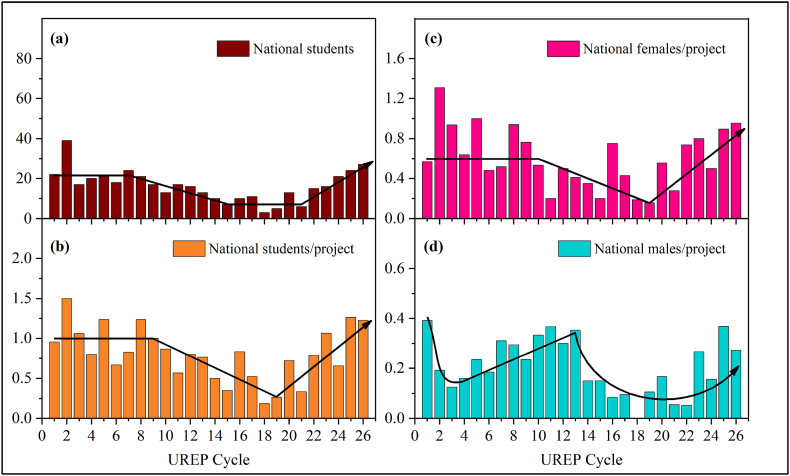
NUG students from QU in STEM disciplines over the First 26 Cycles of UREP. (a) Absolute numbers of national students (b) national students per project (c) National female students per project (d) National male students per project.

Evaluating the participation trend of the national students in STEM disciplines, Fig. 4c–d shows a declining participation rate for both male and female NUG students. Drawing on this study's results, the most noteworthy observation, as shown in Fig. 4a and b, is the detrimental rate at which national male participation dropped down to approximately 0.1 student-to-project proportion (i.e., a ratio of 1:5; one national male student in ten projects) during cycles 16 to 22. Similarly, female national participation also revealed a declining trend during the middle cycles of UREP, with the national female student-to-UREP project rate going as low as 0.16. This severe dip in participation started to ameliorate since cycle 22, and ongoing effective measures need to be maintained to stabilize the involvement of national groups in STEM-related disciplines. In this respect, it is inquisitive to note a significant pattern; while the participation ratios of both male and female NUG students in STEM disciplines are critically below the accepted levels, expatriate female participation in STEM disciplines shows progressive growth along with the number of UREP cycles.

**Fig. 5:**
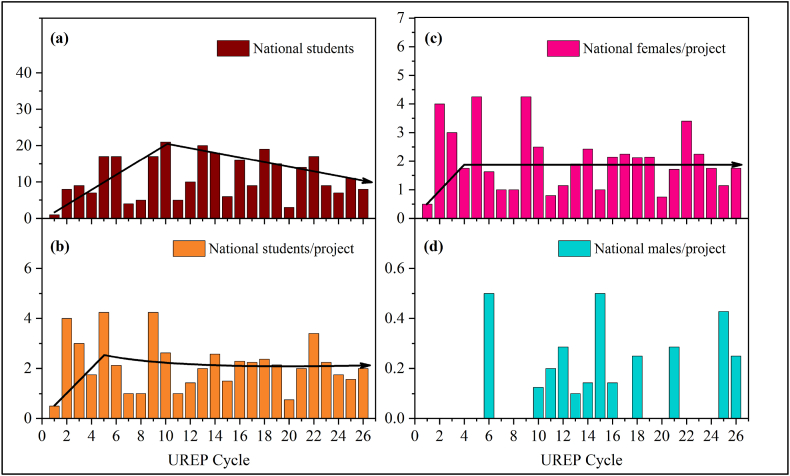
Distribution of QU-NUG students in non-STEM disciplines through the UREP timeline. (a) Absolute numbers of national students (b) National students per project (c) National male students per project (d) National female students per project.

In non-STEM disciplines, the involvement of national students has been stable throughout the UREP cycles, as is shown in Fig. 5a-d. However, constant participation mainly comes from female nationals with a student-to-project rate of more than 1 in almost all cycles of UREP. Demonstrably, male nationals contributed insignificantly with no involvement in most of the cycles, as in Fig. 5 (d). This is a big concern for male national groups, which appear to be on the verge of extinction from UG research if not given the proper attention by authorities.

In summary, it is observed that NUG students have a concerning participation rate in UREP. However, much concern is noted in the participation of male and female NUG student groups in STEM disciplines. These results, derived from the descriptive analyses, lead to asserting the research question that whether this behavior of students towards research is in line with the country's QNV2030.

### 4.2 Significance tests

Independent samples *t*-tests were carried out to measure significant differences between the participation rates (students per project ratio in each UREP cycle) of students based on gender, ethnicity, and discipline. The results of the significant tests are summarized in Table 2.

**Table 2:** Comparison of participation rates of students (student per cycle) in UREP based on ethnic, gender, and discipline-based differences.

Variable	Sub-categories	N	Mean	SD	Min	Max	t	p
Gender	Male	766	1.13	0.36	0.42	1.85	−9.800	0.004
	Female	1689	2.57	0.65	0.96	3.52		
Ethnicity	National	719	1.10	0.34	0.54	1.81	−8.933	<0.001
	Non-National	1736	2.60	0.78	1.42	3.72		
Discipline	STEM	1906	3.64	0.77	2.30	4.94	−0.941	0.246
	Non-STEM	549	3.89	1.06	1.00	5.57		
National Students' Gender	National Male	134	0.19	0.09	0.04	0.38	−10.057	<0.001
	National Female	585	0.90	0.34	0.28	1.61		
National Students' Discipline	STEM National	113	0.80	0.33	0.18	1.50	−6.426	<0.001
	Non-STEM National	313	2.14	1.00	0.5	4.25		

#### 4.2.1 Ethnic differences

The results of the ethnicity-based independent samples t-tests revealed a statistically significant difference between the NUG and expatriate students’ participation rates (*t* (50) = −8.933, *p* < 0.001). Foreign students (*M* = 1.10, *SD* = 0.34) reported significantly greater participation rates than NUG students (*M* = 2.60, *SD* = 0.78).

#### 4.2.2 Gender differences

Results of the independent samples *t*-tests based on gender revealed a statistically significant difference between the participation rate of males and females (*t* (50) = −9.800, *p* = 0.004). Female students demonstrated significantly higher participation rates (*M* = 2.57, *SD* = 0.65) than their male counterparts (*M* = 1.13, *SD* = 0.36). Moreover, assessing gender-based differences between the NUG students, statistically significant differences were found between male and female nationals (*t* (50) = −10.057, *p* < 0.001). Female NUG students (*M* = 0.90, *SD* = 0.34) were reported to have significantly higher participation rates than male NUG students (*M* = 0.19, *SD* = 0.09).

#### 4.2.3 Discipline differences

The discipline-based independent samples *t*-test results did not reveal any statistically significant difference in participation rates between STEM and non-STEM students (*t* (50) = −0.941, *p* = 0.246), even though non-STEM students (*M* = 3.89, *SD* = 1.06) reported slightly higher participation rates than STEM students (*M* = 3.64, *SD* = 0.77). However, statistically significant differences were found between the participation rates of NUG students from STEM and non-STEM disciplines (*t* (50) = −6.426, *p* < 0.001). NUG students in non-STEM disciplines (*M* = 2.14, *SD* = 1.00) demonstrated to have higher participation rates than the NUG students in STEM disciplines (*M* = 0.80, *SD* = 0.33).

### 4.3 Correlation tests

Further, Pearson's correlation tests were performed to analyze the relationship between projects and student participation based on ethnicity, gender, and discipline with the UREP timeline. Correlation tests were implemented to draw the relationship between cycle number, the number of projects, and the number of participants based on gender and ethnicity. The correlation tests were also performed to analyze the effect of the cycle number on the number of national and foreign participants based on gender, being the dependent variances, showcasing diminishing participation from the respective sample. These correlations were drawn for all disciplines in general and also for STEM-based disciplines. The results of the correlation analysis are tabulated in Table 3, Table 4.

**Table 3:** Correlation matrix for students from all disciplines (since UREP cycle 1 to UREP cycle 26).

Variables	UREP Cycle	Projects	Overall Students	Male	Female	National Male	National Female	Non-National Male	Non-National Female
UREP Cycle	1.000								
Projects	−0.054	1.000							
Overall Students	0.702^a^	0.545^a^	1.000						
Male	0.260	0.579^a^	0.656^a^	1.000					
Female	0.742^a^	0.330	0.871^a^	0.200	1.000				
National Male	−0.208	0.593^a^	0.164	0.480^b^	−0.100	1.000			
National Female	−0.182	−0.114	−0.073	−0.361	0.140	−0.179	1.000		
Non-National Male	0.348	0.469^b^	0.679^a^	0.967^a^	0.251	0.240	−0.348	1.000	
Non-National Female	0.795^a^	0.368	0.873^a^	0.346	0.908^a^	−0.022	−0.287	0.390^b^	1.000

a Indicates significance at the 0.01 level.

b Indicates significance at the 0.05 level.

**Table 4:** Correlation matrix for students from STEM disciplines (since UREP cycle 1 to UREP cycle 26).

Variables	UREP Cycle	Projects	Overall Students	Male	Female	National Male	National Female	Non-National Male	Non-National Female
UREP Cycle	1.000								
Projects	−0.164	1.000							
Overall Students	0.582^a^	0.644^a^	1.000						
Male	0.197	0.491^b^	0.674^a^	1.000					
Female	0.638^a^	0.530^a^	0.880^a^	0.242	1.000				
National Male	−0.277	0.565^a^	0.225	0.380	0.050	1.000			
National Female	−0.281	0.317	0.076	−0.199	0.228	0.189	1.000		
Non-National Male	0.296	0.362	0.653^a^	0.965^a^	0.236	0.140	−0.288	1.000	
Non-National Female	0.762^a^	0.400^b^	0.855^a^	0.328	0.912^a^	−0.027	−0.192	0.360	1.000

a Indicates significance at the 0.01 level.

b Indicates significance at the 0.05 level.

For the overall students in UREP (see Table 3), a positive correlation with considerable statistical significance was found between the number of students and the UREP cycle (*r* = 0.702, *p* < 0.05, two-tailed). Pearson's r for the relation depicts a strong to very strong correlation between the variables. This demonstrates the increase in participation of students over the years of the UREP. However, this increase is due to the rise in the number of projects in the UREP, which can be confirmed given the positive correlation between projects and overall students (*r* = 0.545, *p* < 0.05, two-tailed), depicting a moderate to strong correlation with a considerable statistical significance.

Among the students, female students correlated positively with the UREP cycle (*r* = 0.742, *p* < 0.05, two-tailed), showing a strong to very strong correlation. However, male students did not correlate significantly with the UREP cycle. Further, among the female students, the expatriates accounted for the increase. This was demonstrated through a statistically significant and positive correlation between expatriate females and the UREP cycle (*r* = 0.795, *p* < 0.05, two-tailed), which described a strong to very strong correlation. The concern remains for the national male (*r* = −0.208, *p* > 0.05, two-tailed) and female NUG students (*r* = −0.182, *p* > 0.05, two-tailed) who revealed a negative correlation with the UREP cycle, though statistically insignificant. Hence, expatriate student participation can be attributed to the previous positive correlation of overall students with the UREP cycle. However, an interesting finding of the correlation analysis was that male NUG students indicated a positive correlation with the number of projects (*r* = 0.593, *p* < 0.05, two-tailed). This relationship translated to a moderate to strong correlation between the variables, with substantial significance. This implies that there is a valid possibility that the participation of national students can be improved by increasing the number of projects in UREP.

The correlation test results in STEM disciplines signal a similarly diverse pattern of student participation in UREP (see Table 4). The number of STEM students and the UREP cycle was found to have a statistically significant positive correlation (*r* = 0.582, *p* < 0.05, two-tailed). The strength between the variables depicted by Pearson's r indicated a strong to very strong correlation. This shows that STEM students' participation increased over the years of the UREP. However, though this increase may be due to its strong correlation with the number of projects (*r* = 0.644, *p* < 0.05, two-tailed), it should be noted that among the STEM students, the expatriate female students contributed to the increase in overall numbers (*r* = 0.762, *p* < 0.05, two-tailed). On the other hand, national students exhibited negative correlations, though with insignificance (*r* = −0.277 for males and *r* = −0.281 for females). This decline of national students in STEM was at a higher rate than their respective participation in all disciplines.

## 5 Discussion

Developing a research culture that can genuinely contribute to establishing a knowledge-based economy needs dedicated and qualified human capital. Although Qatar has allocated huge investments to attract and instigate researchers from the region and around the world, the country needs to build its national capacity to be able to develop a sustainable future. Not surprisingly, the country is increasingly strengthening its profile as an education hub, elevating its higher education standing by setting up diverse International Branch Campuses (IBCs) from leading world-class universities in addition to its pioneering educational cradle, QU [39].

At the core of this path-breaking reformation lies the fundamental mission to achieve sustainable human development, which is central to establishing a knowledge-based economy in alignment with the goals of QNV2030. Indeed, one of the research initiatives directed at UG students is the UREP, which intends to develop and enhance a research culture among university students. However, the statistical interpretation of the UREP data offered in the present study shows a lack of participation among national students, specifically in STEM-related disciplines. In particular, male NUG students show a worrying trend, with significantly lower involvement in UREP. This lack of participation from NUG students can be linked to the low interest of national students in science disciplines, as indicated in previous literature [10,11,[24], [25], [26], [27], [28]]. A similar study by Kharraz et al. [40] from Saudi Arabia reported lack of time, lack of formal research courses, and lack of supervising mentors as the major barriers preventing medical NUGs from undergraduate research experiences. These factors could also apply to this study. Moreover, other factors such as limited awareness of available research opportunities and how to access them, language barriers, or lack of mentorship could also contribute to the lack of participation of NUG students. More profound research studies (surveys and interviews) examining the perception of national students (especially males) are required to thoroughly understand their lack of participation in UREPs.

However, an interesting finding was that national females showed much higher participation than national males. While this is a positive finding indicating that national females do not face any cultural or societal barriers in UG research in Qatar, it also brings concern towards the underrepresentation of national males in UG research. This finding is supported by previous reports in the literature where males were found to be less interested in STEM due to being attracted to occupations in the public sector [11,41].

These gender and ethnicity differences are a crucial concern for UREP and other endeavors that seek to meet the national goals targeting national capacity building and sustainable development. However, despite these efforts, the minimal levels of national participation point to the lack of the role of institutions in enhancing research awareness among students. Indeed, institutional measures are needed to foster and augment student attitudes toward research and graduate studies [42].

To boost NUG students' participation in research programs, the institutional administrations responsible for R&D should dive into the rationale behind students’ lack of awareness of such research programs [43]. For example, factors likely to influence pre-university student attitudes, including scholastic and non-scholastic settings, also need to be explored, particularly in stages where students transition from secondary schooling to tertiary education [44,45].

Apart from faculty interaction, higher education institutions must examine the role of student's personal attributes and social and cultural influences in determining their research-related attitudes. Indeed, evidence confirms that emotional constructs hold a significant impact in molding students' attitudes toward long-term engagement in learning. For example, work done in Qatar shows the key role of emotion in triggering student interest in STEM fields of study in general [46,47]. These and other studies provide insights into the effect of sub-factors like cultural values that determine UG students' perceptions, especially in Arab countries, where cultural significance is evident [44].

The importance of informal learning experiences in retaining students’ positive attitudes toward STEM UG programs has also been highlighted in various research studies [45,48,49]. These studies conclude that involving students in informal activities is essential to generate their long-term interests in STEM majors and careers. This has proved to be a promising strategy to retain students in STEM-related fields, therefore developing the social and academic capital that contributes to STEM persistence.

Previous studies have also employed gender-biased observations and demographic variables in constructing different perspectives among young UG students [10,46]. Despite the dominance of female NUG student participation in UREP compared to that of their male counterparts, it is yet unknown how many of these students will get into graduate schools or land a job in their respective fields, thereby addressing the requirements of national capacity-building. Moreover, assessing the program's impact on the labor market in Qatar is crucial, as this will enhance participants' work-skill sets. Exploring the program's potential impact can open channels that will eventually attract investments from private-sector agencies, companies, and organizations toward enhancing and supporting a research-driven culture.

As the first study of its kind in the Middle East, this research has far-reaching implications for educators and policymakers in adjacent and other developing countries. It elucidates the value of STEM education as well as the potential benefits of REPs for human capacity building in comparable situations. Most importantly, this study has major implications for educators and policymakers in developing measures to increase male national participation in STEM-related research experiences. Furthermore, there is a vital need for specific measures to stimulate research participation, particularly in STEM subjects, in order to establish a knowledge-based economy in nations such as Qatar. Given the variations in participation rates, REP resource distribution and funding should consider the individual requirements and constraints that different student groups confront to maximize their influence on capacity building. Finally, the study emphasizes the necessity of collaboration among academic institutions, funding agencies, and government organizations to foster UG research engagement and support the aims of establishing a knowledge-based economy. These insights can guide future activities to increase UG research engagement and encourage knowledge-based economic development in Qatar and beyond.

## 6 Limitations

The research undertaken in this study provides essential insights; however, certain limitations must be acknowledged. While the study relies exclusively on quantitative data and statistical analysis, qualitative data, such as interviews or questionnaires, could provide more in-depth insights into the causes of the observed trends in research participation. Furthermore, the study did not investigate the contextual elements impacting research participation, such as institutional culture, financial support, or faculty mentorship, all of which can substantially impact students' decisions to participate in research. Finally, because the study is based on data from a single year, it may miss any changes or advancements in research involvement trends that have occurred after that period. Recognizing these limitations is critical for fully comprehending the breadth and implications of this research. Future studies could address some of these limitations to provide a more comprehensive understanding of UG research in Qatar.

## 7 Concluding remarks and outlook

UREP has so far stimulated a broad array of UG research openings, supplementing existing UG research opportunities available at different academic institutions and contributing to workforce training by funding research activities beyond those offered via regular coursework. However, the participation trends of NUG students in UREP show concerning rates, indicating that some underlying factors hinder the representation of NUG students in UG research. Moreover, assessing the differences in student participation based on demographics revealed that, among the NUG students, it is the male students who are most affected in their participation. Furthermore, while assessing these differences in STEM disciplines, it was found that NUG students showed meager participation in UG research. These results indicate an alarming situation, which constitutes a challenge to the QNV2030 and Qatar Research, Development and Innovation 2030 (QRDI2030) mandate, highlighting the need to investigate the factors that influence and shape students’ interest or lack of interest in REPs.

Future research should cater to the core problems behind the lack of participation of NUG students and their lack of interest in UREPs. Factors that deserve studying include individual, household, and contextual aspects. Additionally, further research should be conducted to provide insights into the anticipated positive impacts of informal and formal learning programs in producing national RDI talent. Also, future research can enable stakeholders to carry out necessary measures to reverse the trend related to low research interest among NUG students. This can also help higher education management design new policies that foster QNV2030 and QRDI2030 objectives. A key research question that researchers need to address in this regard is: what are the key factors influencing NUG students' interest in participating in UREP? Specifically, are there differences in student participation patterns in STEM-based UREP based on gender? Further research to synchronize universities’ approaches to attract more students would facilitate a comprehensive understanding of the behavioral and institutional barriers that inhibit the transformations needed to achieve more sustainable RDI talent. Understanding these aspects will allow policymakers, educators, and stakeholders to plan and design effective strategies to overcome the current situation.

## Availability of data and materials

Limited data is included in the supplementary file referenced in article. Other data can be provided on reasonable request from the readers.

## Funding

This research did not receive any specific grant from funding agencies in the public, commercial, or not-for-profit sectors.

## CRediT authorship contribution statement

**Zubair Ahmad:** Writing – review & editing, Writing – original draft, Supervision, Project administration, Data curation, Conceptualization. **Mohammad Ammar:** Software, Methodology, Formal analysis. **Nitha Siby:** Resources, Formal analysis, Data curation. **Jolly Bhadra:** Writing – original draft, Validation. **Abdellatif Sellami:** Writing – review & editing, Validation. **Noora J. Al-Thani:** Supervision, Project administration, Conceptualization.

## Declaration of competing interest

The authors declare that they have no known competing financial interests or personal relationships that could have appeared to influence the work reported in this paper.

## List of abbreviations

STEM: Science, Technology, Engineering, and Mathematics
REP: Research Experience Program
UREP: Undergraduate Research Experience Program
QNRS: Qatar National Research Strategy
OAR: Office of Academic Research
QNRF: Qatar National Research Fund
UG: Undergraduate
NUG: National Undergraduate
RDI: Research, Development, and Innovation
GDP: Gross Domestic Product
QRDI: Qatar Research, Development, and Innovation
QNV: Qatar National Vision

## References

[1] Russell S.H., Hancock M.P., McCullough J. (2007). Benefits of undergraduate research experiences. Science.

[2] Jones C.K., Lerner A.B. (2019). Implementing a course-based undergraduate research experience to grow the quantity and quality of undergraduate research in an animal science curriculum. J. Anim. Sci..

[3] UNESCO (2021). http://uis.unesco.org/apps/visualisations/research-and-development-spending/.

[4] Kayan-Fadlelmula F. (2022). A systematic review of STEM education research in the GCC countries: trends, gaps and barriers. International Journal of STEM Education.

[5] Sellami A., Arar K., Sawalhi R. (2022).

[6] Tan T., Al-Khalaqi A., Al-Khulaifi N. (2014). Sustainable Development: an Appraisal from the Gulf Region.

[7] Sellami A. (2017).

[8] Baig S.D., Hussain S.I., Qaraqe K.A. (2014). 2014 IEEE Global Engineering Education Conference (EDUCON).

[9] Mohtar R.H. (2015). Opportunities and challenges for innovations in Qatar. Muslim World.

[10] Said Z. (2018). Assessing the science interest, attitude, and self-efficacy of Qatari students at the preparatory, secondary, and university levels. Eurasia J. Math. Sci. Technol. Educ..

[11] Sellami A. (2016). Factors shaping Qatari students' interest in STEM, business or public sector careers. Eurasia J. Math. Sci. Technol. Educ..

[12] Feldman A., Divoll K., Rogan‐Klyve A. (2009). Research education of new scientists: implications for science teacher education. J. Res. Sci. Teach.: The Official Journal of the National Association for Research in Science Teaching.

[13] Sadler T.D. (2010). Learning science through research apprenticeships: a critical review of the literature. J. Res. Sci. Teach.: The Official Journal of the National Association for Research in Science Teaching.

[14] Seymour E. (2004). Establishing the benefits of research experiences for undergraduates in the sciences: first findings from a three‐year study. Sci. Educ..

[15] Lopatto D. (2003).

[16] Ishiyama J. (2001). Undergraduate research and the success of first generation, low income college students. Counc. Undergrad. Res. Q..

[17] Hunter A.-B. (2010).

[18] Bleicher R.E. (1996). High school students learning science in university research laboratories. J. Res. Sci. Teach.: The Official Journal of the National Association for Research in Science Teaching.

[19] Goodlad S. (1998). Research opportunities for undergraduates. Stud. High Educ..

[20] Burgin S.R., McConnell W.J., Flowers A.M. (2015). I Actually Contributed to Their Research': the influence of an abbreviated summer apprenticeship program in science and engineering for diverse high-school learners. Int. J. Sci. Educ..

[21] Estrada M., Hernandez P.R., Schultz P.W. (2018). A longitudinal study of how quality mentorship and research experience integrate underrepresented minorities into STEM careers. CBE-Life Sci. Educ..

[22] Rivers R. (2020). The NIDDK high school short-term research experience for underrepresented persons. Ethn. Dis..

[23] Fakayode S.O. (2014). Promoting undergraduate STEM education at a historically black college and university through research experience. J. Chem. Educ..

[24] Mustafa S.A.-A. (2018).

[25] Kan’an A., Osman K. (2015). The relationship between self-directed learning skills and science achievement among Qatari students. Creativ. Educ..

[26] Lee S. (2016). What motivates and engages students in the education process--an examination of Qatari students' mindset and attitudes toward going to school, learning, and future aspirations. J. Educ. Learn..

[27] Nasser R., McInerney D. (2016). Achievement-oriented beliefs and their relation to academic expectations and school achievement among Qatari students. Educ. Psychol..

[28] Mukhalalati B., Ashour M., Al Noami A.E. (2020). Examining the motivations and future career aspirations of Qatari pharmacy students and alumni: a case study. Currents in Pharmacy Teaching and Learning.

[29] Said R., Kaba A. (2011). 2011 IEEE Global Engineering Education Conference (EDUCON).

[30] Al-Arifi M.N. (2019). Attitudes of pharmacy students towards scientific research and academic career in Saudi Arabia. Saudi Pharmaceut. J..

[31] Abu-Zaid A., Alnajjar A. (2014). Female second-year undergraduate medical students' attitudes towards research at the College of Medicine, Alfaisal University: a Saudi Arabian perspective. Perspectives on Medical Education.

[32] Alsayed N. (2012). Research practices and publication obstacles among interns at king abdulaziz university hospital, jeddah, Saudi Arabia, 2011–2012. J. Egypt. Publ. Health Assoc..

[33] Fund Q.N.R. (2021). https://mis.qgrants.org/Public/AwardSearch.aspx.

[34] Newsome M.L. (2022). The effect of gender and STEM/non-STEM disciplines on remote learning: a national study of undergraduates in Qatar. Electron. J. e Learn..

[35] Emerson R.W. (2015). Causation and Pearson's correlation coefficient. J. Vis. Impair. Blind. (JVIB).

[36] Akoglu H. (2018). User's guide to correlation coefficients. Turkish journal of emergency medicine.

[37] Razali N.M., Wah Y.B. (2011). Power comparisons of shapiro-wilk, Kolmogorov-smirnov, lilliefors and anderson-darling tests. Journal of statistical modeling and analytics.

[38] Shapiro S.S., Wilk M.B. (1965). An analysis of variance test for normality (complete samples). Biometrika.

[39] Weber A.S. (2014). Handbook of Research on Higher Education in the MENA Region: Policy and Practice.

[40] Kharraz R. (2016). Perceived barriers towards participation in undergraduate research activities among medical students at Alfaisal University—College of Medicine: A Saudi Arabian perspective. Medical teacher.

[41] Abdulwahed M. (2013). 2013 IEEE Global Engineering Education Conference (EDUCON).

[42] Dika S.L., D'Amico M.M. (2016). Early experiences and integration in the persistence of first‐generation college students in STEM and non‐STEM majors. J. Res. Sci. Teach..

[43] Ehrenberg R.G. (2010). Analyzing the factors that influence persistence rates in STEM field, majors: introduction to the symposium. Econ. Educ. Rev..

[44] Khalifa B. (2016). Qatar Foundation Annual Research Conference Proceedings.

[45] Soldner M. (2012). Supporting students' intentions to persist in STEM disciplines: the role of living-learning programs among other social-cognitive factors. J. High Educ..

[46] Sellami A. (2017). A path analysis of student interest in STEM, with specific reference to Qatari students. Eurasia J. Math. Sci. Technol. Educ..

[47] Said Z. (2016). Science education reform in Qatar: progress and challenges. Eurasia J. Math. Sci. Technol. Educ..

[48] Habig B. (2020). An informal science education program's impact on STEM major and STEM career outcomes. Res. Sci. Educ..

[49] van Aalderen-Smeets S.I., Walma van der Molen J.H., Xenidou-Dervou I. (2019). Implicit STEM ability beliefs predict secondary school students' STEM self-efficacy beliefs and their intention to opt for a STEM field career. J. Res. Sci. Teach..

