# Supplementary material for: Do research experience programs promote capacity building in Qatar: Investigating the trend and participation differences
Source: Heliyon. 2023 Nov 4;9(11):e22071. doi: 10.1016/j.heliyon.2023.e22071 (PMC10663928; doi:10.1016/j.heliyon.2023.e22071)
Supplement: Supplementary file 1 [file mmc1.docx]

**Table S1.** The division of various sub-disciplinary fields under each primary research area.

| **Primary Research Areas** | **1. Engineering & Technology** | **2. Medical and Health Sciences** | **3. Natural Sciences** | **4. Social Sciences** | **5. Humanities** | **6. Agricultural Sciences** |
| --- | --- | --- | --- | --- | --- | --- |
| **Sub Disciplines** | Environmental Engineering | Basic Medicine | Biological Sciences | Economics and Business | Arts (Arts, History of Arts, Performing Arts, Music) | Agricultural biotechnology |
|  | Chemical Engineering | Clinical Medicine | Chemical Sciences | Educational Sciences | History and Archaeology | Agriculture, Forestry, and Fisheries |
|  | Civil Engineering | Health Sciences | Computer and Information Sciences | Law | Languages and Literature | Other agricultural sciences |
|  | Electrical, Electronic, and Information Engineering | Medical Biotechnology | Earth and Related Environmental Sciences | Media and Communications | Other humanities | Veterinary science |
|  | Environmental Biotechnology | Other Medical Sciences | Mathematics | Other social sciences | Philosophy, Ethics, and Religion | - |
|  | Environmental Engineering | - | Other Natural Sciences | Political Science | Social and Economic Geography | - |
|  | Industrial Biotechnology | - | Physical Sciences | Psychology | - | - |
|  | Materials Engineering | - | - | Social and Economic Geography | - | - |
|  | Mechanical Engineering | - | - | Sociology | - | - |
|  | Medical Engineering | - | - | - | - | - |
|  | Nano Technology | - | - | - | - | - |
|  | Other Engineering and Technology | - | - | - | - | - |

**Table S2.** Descriptive statistics of overall students involved in UREPs from 2006 to 2020.

| Variable | Sub-categories | Number of Participants  (N = 4452) | Percentage of Participants | Number of Projects  (N = 1121) | Percentage of Projects |
| --- | --- | --- | --- | --- | --- |
| Ethnicity | Non-National | 3637 | 78.7 | - | - |
|  | National | 985 | 21.3 | - | - |
| Gender | Male | 1639 | 36.8 | - | - |
|  | Female | 2813 | 63.2 | - | - |
| Primary Research Area | Engineering & Technology | 1640 | 36.8 | 377 | 33.6 |
|  | Medical and Health Sciences | 942 | 21.2 | 275 | 24.5 |
|  | Natural Sciences | 732 | 16.4 | 196 | 17.5 |
|  | Social Sciences | 827 | 18.6 | 202 | 18.0 |
|  | Humanities | 254 | 5.7 | 58 | 5.2 |
|  | Agricultural Sciences | 57 | 1.3 | 13 | 1.2 |
| Discipline | SEM related | 861 | 76.8 | 861 | 76.8 |
|  | Non-SEM related | 260 | 23.1 | 260 | 23.2 |
| Institution | Qatar University | 2972 | 66.7 | 711 | 63.4 |
|  | Texas A&M University at Qatar | 566 | 12.7 | 145 | 12.9 |
|  | Weill Cornell Medical College in Qatar | 382 | 8.5 | 122 | 10.9 |
|  | University of Calgary in Qatar | 143 | 3.2 | 24 | 2.1 |
|  | Georgetown University School of Foreign Service in Qatar | 89 | 2.0 | 36 | 3.2 |
|  | Northwestern University in Qatar | 84 | 1.8 | 13 | 1.2 |
|  | University of Doha for Science and Technology (previously known as College of the North Atlantic in Qatar) | 49 | 1.1 | 9 | 0.8 |
|  | Ahmed Bin Mohamed Military College | 60 | 1.3 | 20 | 1.8 |
|  | Virginia Commonwealth University in Qatar | 47 | 1.0 | 14 | 1.2 |
|  | Carnegie Mellon University in Qatar | 37 | 0.8 | 20 | 1.8 |
|  | Hamad Bin Khalifa University | 12 | 0.2 | 3 | 0.3 |
|  | Community College of Qatar | 4 | 0.0 | 1 | 0.1 |
|  | Stenden University Qatar | 3 | 0.0 | 1 | 0.1 |
|  | Unaffiliated or missing* | 4 | 0.0 | 2 | 0.2 |

* These institutions are not currently active
